# Complex Body Wall Closure Defects in Seven Dog Fetuses: An Anatomic and CT Scan Study

**DOI:** 10.3390/ani15142030

**Published:** 2025-07-10

**Authors:** Nieves Martín-Alguacil, José M. Cozar, Luis Avedillo

**Affiliations:** Departmental Section of Anatomy and Embryology, School of Veterinary Medicine, Universidad Complutense de Madrid, 28040 Madrid, Spain; jcozar@ucm.es (J.M.C.); luiavedi@ucm.es (L.A.)

**Keywords:** body stalk anomalies (BSAs), BSA classification, canine, craniofacial malformations, LBWC, non-structural skeletal anomalies, non-structural urogenital anomalies, structural skeletal anomalies, structural urogenital anomalies, amniotic bands

## Abstract

Body stalk anomaly (BSA) is a rare and usually fatal congenital disorder involving severe malformations of the body wall, limbs, spine, and internal organs. This study presents the first documented cases of BSA in seven dogs, offering new insights into how the disorder manifests in animals. The affected fetuses consistently exhibited major anomalies, including large abdominal wall defects, structural spinal abnormalities, and a variety of limb malformations ranging from partial agenesis and meromelia to phocomelia and complete amelia. Structural urogenital anomalies and orofacial clefts were also observed, aligning with similar findings in BSA cases reported in pigs and cats. These findings support the hypothesis of a multifactorial etiology involving early embryonic disruptions, such as abnormal folding of the embryo, rupture of the amniotic membrane, and vascular compromise. The frequent occurrence of abdominal wall defects alongside umbilical cord abnormalities further suggests a shared developmental pathway. This study also highlights the value of veterinary cases in comparative embryology and the need to assess congenital anomalies as part of a broader malformation complex. By expanding the phenotypic spectrum of BSA in domestic animals, this work contributes to a deeper understanding of its pathogenesis and emphasizes the importance of further research into the genetic and environmental factors involved. Such efforts could lead to improved classification and diagnosis of complex congenital malformations, as well as facilitate cross-species comparisons.

## Simple Summary

Body stalk anomaly (BSA) is a rare and severe birth defect that causes significant developmental issues affecting the body wall, spine, limbs, and internal organs. This study describes the first known cases of BSA in seven dogs. The affected puppies exhibited comparable patterns of abnormalities, including large openings in the abdominal wall, spinal deformities, absent or shortened limbs, urinary and reproductive system issues, and facial clefts. These findings suggest that BSA is caused by a combination of genetic, environmental, and developmental factors affecting the embryo in the early stages of pregnancy. Studying these cases in animals could help scientists to better understand how these complex birth defects occur and how they relate to similar conditions in humans.

## 1. Introduction

Complex body wall anomalies (CBWAs) are a group of rare congenital anomalies characterized by severe malformations in the closure of the body wall, affecting the thoracic, abdominal, and sometimes craniofacial regions. These defects can result in the protrusion of internal organs outside the body cavity, leading to significant health challenges. One of the most notable conditions within this group is body stalk anomalies (BSAs). BSAs are complex defects of the body wall in which, in addition to a body wall anomaly, there are structural skeletal abnormalities, and the umbilical cord (UC) is abnormal, absent, or rudimentary, with or without craniofacial abnormalities [1]. The classification of BSA within the broader group of CBWAs is supported by the current classification systems and clinical literature. BSA is recognized as a major type of complex body wall anomalies, often grouped alongside limb–body wall complex (LBWC) and other severe body wall defects [2,3,4,5,6,7]. Classification systems, such as the Martin-Alguacil CBWA classification, include BSA as a specific category within CBWAs, sometimes overlapping with LBWC, spinal, and sternal complex defects [8]. The study proposed a new classification system for BSAs, dividing them into eight types based on combinations of defects in the body wall, the umbilical cord, and the skeleton (spine, sternum, and limbs). In the study, thirty piglets with varying degrees of BSA were analyzed. Most had large ventral wall defects, spinal anomalies, and abnormal UC. Limb defects were always associated with spinal anomalies, leading to the creation of a new classification called the spinal–limb–body wall complex (SPLBWC). Other classifications include the spinal–body Wall Complex (SPBWC), the sternal–body wall complex (STBWC), and the sternal–spinal–body wall complex (SSBWC). Structural anomalies were categorized into spinal, sternal, and limb defects, while non-structural anomalies (e.g., deformities caused by amniotic bands) were excluded [8]. The anomalies were grouped into cranial, abdominal, and cranioabdominal overlapping phenotypes. Most of the piglets showed anomalies in the umbilical cord, such as scattered vessels or a single umbilical artery (SUA). BSA together with an amniotic band disruption complex (ABDC) was also studied in six cats [9]. In this study, structural defects such as spinal and limb anomalies (e.g., arthrogryposis, ectrodactyly, amelia, phocomelia), craniofacial anomalies (e.g., cleft palate, exencephaly) and UC irregularities were documented. In veterinary medicine, the prenatal diagnosis of congenital anomalies such as BSA, AB, and ABDC remains challenging due to limited routine use of high-resolution fetal imaging [10]. However, with advanced ultrasonography, major structural defects—including abdominal wall malformations, spinal deformities, and limb anomalies—may be detectable from approximately day 30 of gestation in such species as the dog [10]. In most cases, definitive diagnosis is made postnatally through examination of stillborn or aborted fetuses, highlighting the importance of integrating prenatal imaging with postmortem findings to improve early detection and understanding of these rare syndromes.

The aim of this study was to use the classification system originally developed for humans and pigs, and recently applied to cats [9], to describe and analyze BSA in dogs and to provide insight into the causes, physical manifestations, and potential management of these abnormalities. The classification system used has been shown to be helpful in diagnosing, investigating, and managing BSA more effectively in both veterinary and human medicine. It highlights the importance of understanding the embryological origins of these anomalies, which are associated with early developmental abnormalities. Knowledge gained from animal models, in this case, the canine model, can improve understanding of similar congenital anomalies in humans, aiding diagnosis, treatment, and research.

## 2. Materials and Methods

This observational study involved the examination of cadavers from seven dog fetuses presenting body wall closure defects. The specimens were provided by licensed veterinarians and obtained in full compliance with the European Union regulations (Directive 2010/63/EU) and the Spanish legislation (RD 53/2013). All cases were analyzed at the Laboratory for the Study of Congenital Malformations (GIMCAD 971005-UCM), part of the Departmental Section of Anatomy and Embryology at the School of Veterinary Medicine, Universidad Complutense de Madrid, Spain.

The cases were sporadically identified during cesarean sections or natural deliveries conducted between 2007 and 2022. The characteristics of these dogs are detailed in Table 1. Maternal health was confirmed through clinical evaluation, which ruled out infectious or contagious diseases. Routine preoperative hematological and biochemical analyses revealed no abnormalities. All dams had received standard vaccinations and antiparasitic treatments. The dog fetuses were delivered at full term, with no visible anomalies in littermates.

Immediately post-delivery, the specimens underwent the following procedure:

- Initial examination and fixation: each fetus was examined macroscopically and then fixed in 5% formaldehyde for preservation.
- Anthropometric measurements: body weight was recorded using a digital precision scale, and body length was measured using a flexible tape.
- Gross anatomical evaluation: standard dissection techniques were employed to assess and document structural malformations across multiple anatomical systems.
- Radiographic imaging: radiographs were taken using a Sedecal (Algete, Madrid, Spain) 20 kW three-phase high-frequency X-ray unit equipped with a Toshiba Rotanode E7239X F 2 mm–1 mm X-ray tube (Otawara, Japan) and an Optimus DR direct digital detector panel.
- Computed tomography (CT) analysis: high-resolution CT scans were performed using a Toshiba Aquilion 64 multislice CT scanner (350 mA, 120 kV, 512 × 512 matrix, 5.5 cm field of view). Images were acquired with a bone reconstruction algorithm (window level 550, window width 2550, slice thickness 0.5 mm, pitch 0.4 mm).
- 3D visualization: CT data were reconstructed in three dimensions to enhance identification and analysis of complex congenital anomalies.

Each case was diagnosed and classified for body wall closure defects (BSA) based on criteria described by Martín-Alguacil et al. in swine and human models [3,7,8]. Classification followed a diagnostic system established for porcine BSA subtypes (types I–VIII). Further subclassification into body wall complex (BWC) syndromes—including SPBWC, SPLBWC, STBWC, and SSBWC—was performed when relevant structural anomalies were present. The term meromelia was used to describe partial limb absence, despite its absence from the official Nomina Embryologica Veterinaria as it best fit several observed phenotypes.

## 3. Results

All of the abnormalities that were observed in the 7 dogs are listed in Table 2.

**Figure 1:**
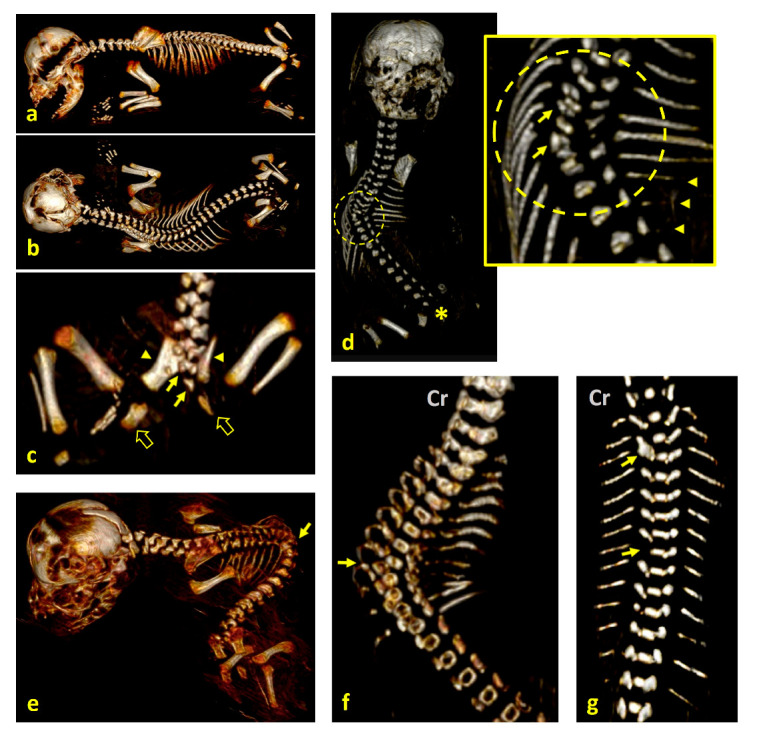
Visualization of spinal structural anomalies on 3D VR (volume rendering) CT scan reconstructions of fetal specimens. The abdominal viscera were digitally removed with imaging software to enhance visibility of the vertebral column and the associated skeletal structures—no physical dissection was performed. (**a**) Lateral view and (**b**) dorsal view of case 1, showing scoliosis; (**c**) dorsal view of the caudal end of the spine and the pelvis highlighting hypoplasia of S1 and S2 (arrows), agenesis of S3 and the caudal vertebrae, hypoplasia of both ilia (arrowheads), malformed ischia (fat arrows), and agenesis of both pubic bones; (**d**) dorsal view of case 2, affected by scoliosis; hemivertebrae at T5–T7 (inset, arrows), hypoplasia of the left ribs 6–8 (arrowheads), agenesis of the left ribs 10–13, and absence of the sacral and caudal vertebrae (asterisk); (**e**) left dorsal view of case 3, showing left lateral retroflexion (extreme scoliosis), with a vertebral block at the point of maximum flexion (arrow), and hemivertebrae at the thoracolumbar junction; (**f**) dorsal view of case 4, with scoliosis and hemivertebrae at T7–T9, corresponding to the apex of spinal curvature (arrow); (**g**) dorsal view of case 6, showing scoliosis with a vertebral block at T2–T3 (upper arrow) and hemivertebra at T8 (lower arrow). Note: Cr, cranial.

**Figure 2:**
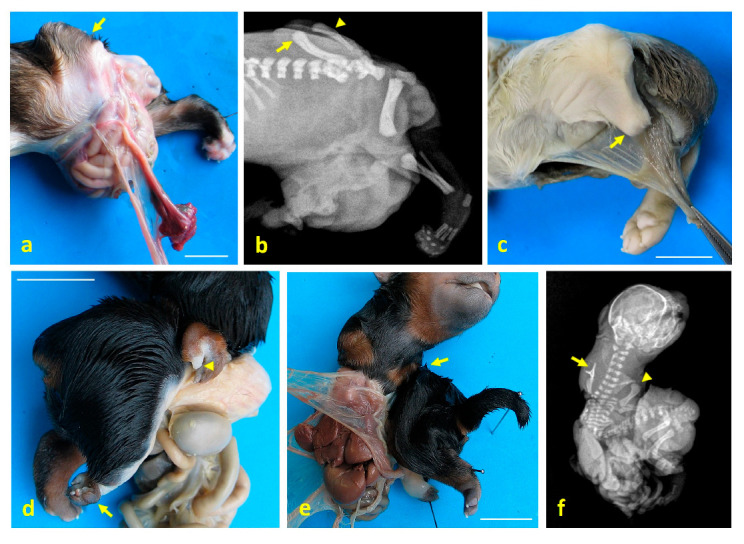
Limb structural anomalies in dog fetuses using digital imaging and radiography. (**a**) Left lateral view of the caudal third of case 1, showing meromelia of the left pelvic limb (arrow); (**b**) lateral X-ray of the same region in case 1, showing misaligned femur (arrow), tibial hypoplasia (arrowhead), and absence of distal skeletal structures; (**c**) caudolateral view of case 2, with meromelia of the left pelvic limb (arrow); (**d**) right ventrolateral view of case 3, showing meromelia of the right thoracic limb (arrowhead) and phocomelia of the right pelvic limb (arrow); (**e**) ventral view of case 3, showing amelia of the left thoracic limb (arrow); (**f**) ventrodorsal X-ray of case 3, showing amelia of the left thoracic limb (arrow) and meromelia of the right thoracic limb (arrowhead). Scale bar: 1 cm.

**Figure 3:**
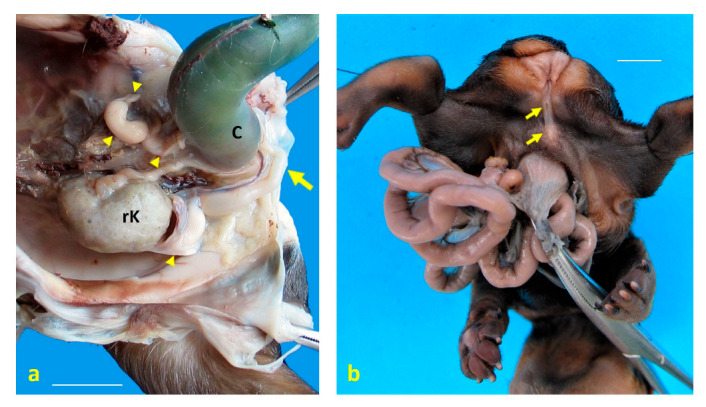
Urogenital structural anomalies observed in dog fetuses. (**a**) Left ventrolateral view of the pelvic cavity in case 1, showing a persistent urogenital sinus (arrow) and genital hypoplasia following caudal retraction of the colon (C). Cystic remnants of paramesonephric (Müllerian) ducts are indicated by arrowheads. A single right kidney (rK) is located in the pelvis, consistent with renal ectopia and agenesis; (**b**) caudoventral view of the perineal region in case 5, revealing anal atresia and complete hypospadias (arrows). Scale bar: 1 cm.

Case 1: a left caudolateral omphalocele was observed, with external exposure of the spleen, liver, gallbladder, pancreas, part of the stomach, and all intestines except the descending colon and the rectum, all covered by a membrane. Scoliosis was noted, along with hypoplasia of S1 and S2, agenesis of S3 and the caudal vertebrae, and hypoplasia of both the ilium and the ischium, with agenesis of both pubes (Figure 1a–c). The umbilical cord was found to contain a single umbilical artery (SUA). The left pelvic limb was affected by meromelia (Figure 2a,b). Additional anomalies included anal atresia (AA), persistent urogenital sinus, genital hypoplasia, and left renal agenesis (Figure 3a). Testes were identified in the inguinal ring.

Case 2: a left caudolateral abdominoschisis was present, resulting in eventration of a large portion of the small intestine. UC vessels were found scattered and not forming a tubular UC. Scoliosis was observed, along with hemivertebrae at T5–T7 (Figure 1d), hypoplasia of the left ribs 6, 7, and 8, and agenesis of the left ribs 10 to 13. Agenesis of the sacral and caudal vertebrae was also noted. The left pelvic limb was affected by meromelia (Figure 2c). Testes were found to be intra-abdominal. Hypoplasia of the genital tract and its associated organs was identified.

**Figure 4:**
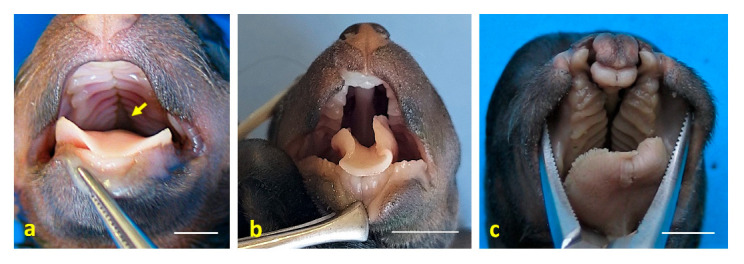
Craniofacial anomalies observed in dog fetuses. (**a**) Dorsal view of the oral cavity roof in case 3, showing secondary palatoschisis (arrow); (**b**) dorsal view of the oral cavity in case 4, also showing secondary palatoschisis. Note the nasal morphology, which resembles that of a mouse; (**c**) dorsal view of the oral cavity in case 6, displaying cheilognathopalatoschisis. Scale bar: 1 cm.

Case 3: a right lateral abdominoschisis was identified, with external exposure of the kidneys, spleen, liver with gallbladder, pancreas, part of the stomach, and all intestines except the descending colon and the rectum. The UC was not identified. Left lateral retroflexion with extreme scoliosis was observed (Figure 1e). A vertebral block was present at the point of maximum spinal flexion, along with hemivertebrae at the thoracolumbar junction. Amelia of the left thoracic limb and meromelia of the right thoracic limb were noted, as well as phocomelia of the right pelvic limb with arthrogryposis (Figure 2d). Secondary palatoschisis (Figure 4a), bilateral renal hypoplasia, and genital hypoplasia were also documented. Testes were found to be intra-abdominal.

**Figure 5:**
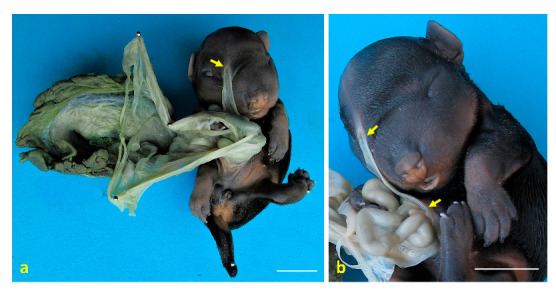
Congenital amniotic adhesion in a dog fetus. (**a**) Ventral view of case 5 with the placenta, showing an amniotic adhesion at the frontal region (arrow); (**b**) close-up view of case 5, highlighting the amniotic membrane adhered to the frontal region, with visible remnants of the amnion attached to the external nares (arrows). Scale bar: 1 cm.

Case 4: omphalocele was observed, with eventration of a large portion of the small intestine (jejunum). Scoliosis with hemivertebrae at the T7–T9 level was noted (Figure 1f), corresponding to kyphosis. Arthrogryposis of the pelvic limbs was present, along with anal atresia (AA). Secondary palatoschisis and a snout resembling that of a rodent were also identified (Figure 4b). Hypoplasia of the genital tract and organs was observed, and testes were found to be intra-abdominal. An amniotic band adhesion was present in the right ventrolateral frontal region, with remnants of the amnion attached to the nostrils (Figure 5a,b).

Case 5: omphalocele with eventration of jejunal loops was identified. Arthrogryposis of the cervical and first thoracic vertebrae was observed, along with agenesis of the sacrum and the caudal vertebrae. Arthrogryposis of both pelvic limbs was present, with the plantar surfaces of both extremities oriented medially. Anal atresia (AA) was noted, and intra-abdominal testes were found, as well as complete hypospadias (Figure 3b).

Case 6: omphalocele and eventration of jejunal loops were observed. Mild scoliosis was present, with a vertebral block at the T2–T3 level and a hemivertebra at T8 (Figure 1e,g). Arthrogryposis of the right thoracic limb was noted. Cheilognathopalatoschisis was also identified (Figure 4c). Testes were found to be intra-abdominal.

Case 7: omphalocele and eventration of jejunal loops were observed. Moderate scoliosis and arthrogryposis of the pelvic limbs were present, with the plantar surfaces of both extremities oriented dorsally. Hydrocephalus was also noted. Testes were found to be intra-abdominal.

Three dogs (cases 1–3) were diagnosed as BSA type III and SPLBWC, two cases (4, 5)—as BSA type IV and SPBWC, and two cases (6, 7)—as BSA type VIII and SPBWC.

**Figure 6:**
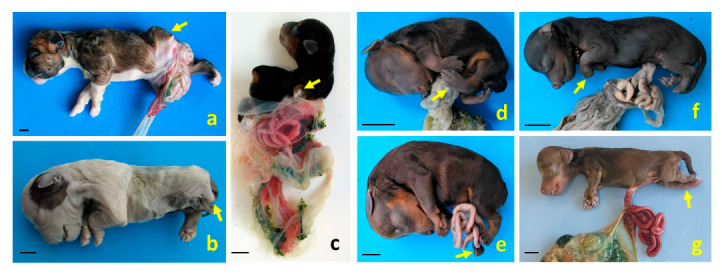
Structural anomalies and body wall defects diagnosed as different BSA types in dog fetuses. (**a**,**b**) Left lateral views of cases 1 and 2, respectively, both exhibiting defects in body wall closure and meromelia of the left pelvic limb (arrows); (**c**) right dorsolateral view of case 3, showing the full extent of the amnion. This case presents severe scoliosis, amelia of the left thoracic limb (arrow), meromelia of the right thoracic limb, and phocomelia of the right pelvic limb. Cases 1–3 were diagnosed as BSA type III and spinal body wall complex (SPLBWC); (**d**,**e**) left lateral views of cases 4 and 5, showing pelvic limb malpositioning due to arthrogryposis (arrows), diagnosed as BSA type IV and spinal body wall complex (SPBWC), respectively; (**f**,**g**) left lateral views of cases 6 and 7. Case 6 presents omphalocele and arthrogryposis in the thoracic limbs (arrow); case 7 shows omphalocele and arthrogryposis in the pelvic limbs (arrow). These cases were diagnosed as BSA type VIII and SPBWC, respectively. Scale bar: 1 cm.

The different BSA types diagnosed are shown in Figure 6.

## 4. Discussion

LBWCs and BSAs are rare and severe congenital malformations that fall within the spectrum of complex body wall defects (CBWDs) [7,8,9]. They are characterized by large ventral body wall closure defects, typically abdominoschisis or thoracoabdominoschisis, accompanied by spinal deformities, limb anomalies, and umbilical cord abnormalities [7,8,9]. A hallmark feature of both LBWCs and BSAs is abdominoschisis [11,12,13,14], which may be midline or lateral, and is often associated with evisceration of abdominal organs [15,16,17,18].

The pathogenesis of these anomalies remains uncertain, though several theories have been proposed. These include early amnion rupture, vascular disruption and intrinsic embryonic dysgenesis, particularly involving failures in embryonic folding and body wall placode development [19,20]. Based on Paul Portal’s initial description of LBWC in 1685 [21], Van Allen et al. (1987) [22] defined this condition as the presence of at least two of the following: exencephaly or encephalocele with facial clefts, thoraco- and/or abdominoschisis, and limb anomalies. Although BSAs and LBWCs are phenotypically similar, BSA is typically characterized by significant body wall defects, as well as spinal and genitourinary abnormalities, anal atresia, and an absent or short umbilical cord [7,8,9,11,12,13,14]. Recent veterinary studies have highlighted the presence of these anomalies in pigs, where LBWCs and BSAs have been observed and classified using detailed anatomical and embryological criteria [7,8]. In these models, LBWC is considered a subset of BSAs, particularly when structural limb defects are present. This has led to the development of more refined classification systems, including eight BSA types, as well as additional categories such as the spinal–limb–body wall complex (SPLBWC) [8]. These systems account for the frequent co-occurrence of spinal and limb anomalies.

In BSAs, the umbilical cord is consistently affected, frequently being either absent or rudimentary, with the umbilical vessels being dispersed in the amniotic membrane rather than forming a discrete cord structure [23,24,25,26]. These features distinguish abdominoschisis in LBWCs and BSAs from isolated abdominal wall defects, such as gastroschisis and omphalocele [15]. Gastroschisis usually presents as a paraumbilical defect on the right side of the body with exposed abdominal contents and no covering membrane [27]. In contrast, omphalocele is a midline defect where herniated organs are enclosed in a membranous sac, through which the umbilical cord passes [28]. By contrast, abdominoschisis in LBWC/BSA is often extensive and is associated with severe spinal deformities, limb anomalies, and abnormal or absent umbilical cords. While gastroschisis and omphalocele may be surgically correctable with favorable outcomes, LBWCs and BSAs are associated with multisystem anomalies and a poor prognosis [29]. Although the term “abdominoschisis” is not commonly cited in standard veterinary embryology textbooks, it is well-established in the human medical literature, particularly in the context of LBWCs and BSAs [11,12,13,14,15,16,17,18,19,20,21,22,23,24,25,26].

In veterinary medicine, the term “schistosomus reflexus” (SR) has historically been used to describe severe congenital malformations that share many features with BSAs and LBWCs, including spinal retroflexion, ventral body wall defects, and limb anomalies [30]. SR has been reported in ruminants, pigs, and cats [31,32,33], but is not recognized in human medicine, where similar cases fall under the BSA classification. This reflects a divergence in classification systems: SR serves as a broad, descriptive term in veterinary contexts, whereas human medicine has adopted more structured, pathogenesis-based terminology [3]. Given the anatomical and developmental similarities, this study advocates for adopting BSAs as a more precise comparative framework in veterinary pathology. Standardizing terminology across species could improve diagnostic clarity, enhance comparative pathology, and contribute to a deeper understanding of congenital developmental disorders.

All cases in this study exhibited incomplete closure of the abdominal wall. In dogs, the amniotic sac forms between gestational days 18 and 21, with body wall closure occurring between days 19 and 35. Disruptions during this critical period, particularly between days 18 and 30, can lead to the formation of amniotic bands, which may entangle fetal structures and cause deformities [27].

In this series of seven cases of congenital abdominal wall defects, we observed a variety of presentations, including four midline omphaloceles, one caudolateral omphalocele, one left caudolateral abdominoschisis, and one right lateral abdominoschisis. The midline omphaloceles displayed the classic appearance of herniated abdominal contents enclosed in a membranous sac at the umbilicus. In contrast, the caudolateral omphalocele was displaced, likely due to the absence of pelvic bones, allowing the sac to shift caudolaterally. By contrast, the abdominoschisis cases, particularly case 3, exhibited extensive lateral wall defects without a protective sac and were associated with additional anomalies.

Comparative studies have shown that BSA phenotypes vary across species [3,8,9]. In cats, one case involved an abdominal presentation, while three involved cranioabdominal defects [9]. In pigs, 11 cases were cranial, 11 were abdominal, and seven were cranioabdominal [8]. As reported in the present study, three cases of BSA were observed in dogs, exhibiting abdominal defects, and four cases showed cranioabdominal involvement.

The presence of a single umbilical artery (SUA) in case 1 can be explained by early disruptions in embryonic development. SUA is a common umbilical cord anomaly that is often associated with congenital malformations of the cardiovascular, renal, and body wall systems [34,35]. In this study, a SUA was identified in a dog with CBWDs, which is consistent with previous reports in other species, such as piglets, where SUA and unilateral umbilical artery hypoplasia have been observed alongside CBWDs [7]. Developmentally, SUA is generally attributed to the failure of one umbilical artery to form (aplasia), or to its early regression (atrophy), during embryogenesis [35]. These vascular anomalies are often linked to broader morphogenetic disruptions, particularly in conditions such as BSAs and Cantrell syndrome (CS), where impaired embryonic folding and ventral wall closure affect umbilical cord formation. In CS, SUA may indicate a broader midline developmental defect [3,7,8,9,36]. In veterinary literature, SUA has been observed in dogs, particularly in association with high-risk pregnancies and fetal anomalies. Doppler studies in canine pregnancies have shown that abnormalities in umbilical artery flow can be indicative of compromised fetal development [37]. Similar findings in piglets [7,8,9] and humans [3] further support the idea that SUA and amniotic bands are markers of early embryological insult across species.

Structural skeletal defects were a consistent feature of the cases studied, with spinal, rib, and limb anomalies observed in multiple individuals. Vertebral congenital malformations are well-documented in brachycephalic breeds such as French bulldogs, English bulldogs, and pugs and are often associated with genetic mutations, such as those in the DVL2 gene [38]. In the context of BSAs, the co-occurrence of spinal and other systemic malformations suggests a shared developmental origin, highlighting the significance of early embryonic events in shaping the complex phenotypes observed in these cases.

Limb anomalies such as amelia (the complete absence of a limb), meromelia (the partial absence of a limb), and phocomelia (absence or severe underdevelopment of the proximal limb segments, with the distal limb attached close to the trunk) were also observed. Limb meromelia and arthrogryposis (joint contractures) were classified as non-structural defects, phocomelia and amelia—as structural defects [3,7,8]. These skeletal and limb abnormalities are believed to result from disruptions to early embryonic development, which may be influenced by restricted fetal movement or amniotic bands. The limb malformations observed in the studied cases reflect a wide spectrum of developmental anomalies, ranging from the partial absence of limb structures to complete limb agenesis [39]. Although partial absence of limbs has been documented in various species, including dogs [39,40,41], cases of true amelia in canines are rare [42,43]. In the present study, the presence of amelia, meromelia, and phocomelia in case 3 further highlights the severity of the developmental disruption associated with BSAs. In human medicine, amelia frequently occurs alongside other major congenital anomalies, such as anencephaly, cleft lip, and abdominal wall defects [3,44]. Similar associations have been reported in other animal species, including pigs and cats [7,8,9], where amelia often co-occurs with multiple congenital anomalies.

Structural genitourinary defects. AA is a congenital malformation resulting from failure of the urorectal fold to separate from the primitive cloaca or the opening of the anal membrane [38,45]. Persistent urogenital sinus is a rare congenital anomaly in dogs, resulting from incomplete separation of the urogenital and rectal tracts during embryogenesis, and is often associated with other malformations such as anal atresia, rectourethral fistula, genital hypoplasia, and renal agenesis [38].

Hypospadias is a congenital condition in which the urethra does not terminate normally at the glans penis [38]. It is caused by a combination of genetic and environmental factors, and the severity depends on the location of the urethral opening. This can occur in the shaft of the penis or in the perineal region [46]. Associated malformations include cryptorchidism, penile aplasia or hypoplasia, a bipartite scrotum, testicular agenesis, hermaphroditism, and anal atresia [47]. In the literature, hypospadias appears to be an uncommon malformation; [46] reported an incidence of 0.05% in dogs, with German shepherds being the most prevalent breed. In humans, the high heritability of this malformation indicates a strong hereditary component [48], but studies focusing on the genetics of the condition in dogs are scarce.

Craniofacial anomalies. Orofacial clefts refer to abnormal clefts in orofacial structures resulting from incomplete tissue fusion during embryonic development [38] and have been observed in several mammalian species, including dogs [49]. These clefts are classified into cleft lip, cleft palate, and cleft lip and palate [50]. In humans, approximately 70% of orofacial cleft cases occur in isolation, without cognitive or craniofacial abnormalities, and these cases are referred to as non-syndromic orofacial clefts [51]. Orofacial clefts, such as cleft lip and cleft palate, are often associated with dysraphic malformations, which result from defects in the closure of embryonic structures. In humans, for example, cleft lip has been linked to omphalocele, a defect in the closure of the abdominal wall [44]. Notably, cleft lips have also been observed in cases of BSAs in pigs [7,8] and cats [9], and, in the present study, in dogs. This further supports the idea that these complex malformations have a shared developmental origin. These associations highlight the need for further research to better understand the links between orofacial clefts and dysraphic malformations.

The canine cases showed a variety of congenital anomalies associated with amniotic band disruption (ABDC) [1,9]. However, the presence of an amniotic band was only observed in case 4. We believe the BSA cases in this study reflected a spectrum of complex congenital anomalies caused by mechanical and vascular effects of amniotic bands [52,53,54,55,56]. The variability in malformations is largely due to the timing of disruption during development. Early disruptions (organogenesis) can result in agenesis or incomplete formation of limbs or organs (e.g., amelia in case 3, renal agenesis in case 1), while later disruptions may cause constriction or amputation of the already formed structures (e.g., meromelia in case 3). Some cases suggest that exposed or abnormal embryonic facial tissue (mesenchyme) is more susceptible to adhesion by fetal parts or amniotic bands, leading to complex craniofacial clefts [57]. Facial clefts often co-occur with amniotic bands, which can physically interfere with normal development [56,58,59,60]. Experimental models confirm that constricting bands can induce both soft tissue and bony clefts. Certain facial structures, such as the nasal processes, may be more prone to band attachment, leading to atypical clefts [55,56]. The co-occurrence of limb and craniofacial anomalies, as observed in case 3, supports a shared disruptive mechanism [55,56,59,60]. Although amniotic band entrapment is a common cause, the phenotypic outcome depends on the developmental stage at which the disruption occurs—whether it impairs formation or damages already formed structures.

ABs and BSAs frequently occur together, often resulting in severe and complex fetal malformations, as observed in this study and in other species, including humans [1,3,7,8,9]. It is believed that their association stems from early amniotic membrane rupture, which leads to mechanical and vascular disruptions. Such rupture can cause compression-related defects such as gastroschisis, abdominoschisis, thoracoabdominoschisis, neural tube defects, and scoliosis [1,9,61,62,63,64]. ABDCs and BSAs share overlapping pathogenetic mechanisms, including amniotic or vascular disruption and possible genetic factors [1,9,16,62,63,64]. The timing of the rupture is critical; early ruptures tend to cause severe body wall and internal malformations, whereas later ruptures more often result in isolated limb deformities or amputations [1,9,16,44,65]. BSA cases typically present with a spectrum of anomalies, including body wall defects, limb constrictions or amputations, craniofacial malformations, and internal organ anomalies [16,62,63,65]. While ABDCs without body wall defects and BSAs with extensive anomalies may represent distinct conditions, both may involve amniotic bands, particularly when disruption occurs early in development [1,7,8,9,18,44,63,64].

The AB syndrome and AB sequence within the ABDC are malformative processes explaining some observed anomalies. However, genitourinary defects in cases 1–5 suggest an earlier developmental disruption not accounted for by ABDC mechanisms. Craniofacial clefts in cases 3, 4, and 6, along with amniotic adhesions in the extraembryonic coelom, may have resulted from mesenchymal tissue prone to adhesion. Weinstein et al. [58] described craniofacial clefts with limb–facial adhesions, supporting intrinsic and adhesion-based theories of AB syndrome. Robin et al. [66] reported a case with typical ABDC features plus anomalies inconsistent with the extrinsic model, echoing findings by Guion-Almeida and Richieri-Costa [67]. This suggests a possible unrecognized syndrome and a genetic basis for some ABDC cases, especially those with cleft lip and palate, such as case 4.

While some authors report no gender predilection in LBWCs [68], Martin-Alguacil and Avedillo [7] found a strong female bias in pigs, with 77% (23/30) of the affected piglets being female—82% in the abdominal, 64% in the cranial, and 100% in the cranioabdominal phenotype groups. Similarly, in cats with the cranioabdominal phenotype, two of three cases were female [9]. In contrast, most affected dogs were male, indicating interspecies variability. These findings suggest a potential sex-related influence on BSA manifestation and underscore the importance of considering sex in congenital anomaly research. They also support the value of animal models in the One Medicine approach to improving cross-species health outcomes. Further research is needed to clarify the underlying mechanisms and enhance diagnosis and treatment.

To the best of our knowledge, this is the first report to describe BSAs in dogs. While there are reports of limb malformations in dogs, such as thoracic limb malformations [69], amelia, meromelia [70,71], and arthrogryposis, these have not previously been linked to the wider range of anomalies typically associated with BSA. Similar complex congenital anomalies have been documented in other species, including pigs and cats. The canine cases presented here expand the known phenotypic spectrum of BSAs in veterinary medicine. The presence of both abdominal and cranioabdominal phenotypes alongside detailed skeletal and urogenital anomalies lends weight to the hypothesis that BSAs originate from early embryonic disruptions. Given the scarcity of veterinary-specific studies on BSAs, references to human medical literature were necessary to provide embryological context and comparative insights that support the interpretation of the observed anomalies. By referencing the established literature on veterinary embryology and theriogenology, this study highlights the uniqueness of the canine presentation and contributes to a broader understanding of the developmental mechanisms underlying such anomalies.

## 5. Conclusions

The abnormalities observed in these canine cases emphasize the complex and multifactorial nature of body stalk anomaly (BSA) in veterinary medicine. The occurrence of severe skeletal malformations, such as spinal deformities and limb anomalies, alongside craniofacial defects, hydrocephalus, and urogenital abnormalities emphasizes the wide range of symptoms associated with this condition. These findings support a shift in veterinary diagnostic orientation, moving away from evaluating congenital anomalies in isolation and towards assessing them as part of a broader malformation complex, particularly when multiple defects are present. This integrative approach mirrors trends in human medicine and emphasizes the importance of identifying patterns of associated anomalies in order to better understand their developmental origins.

The diversity of malformations observed is consistent with a multifactorial etiology involving genetic predisposition, environmental influences, and mechanical disruptions during early embryogenesis. The consistent association between abdominal wall closure defects and umbilical cord abnormalities suggests that early rupture of the amniotic membrane may play a central role in pathogenesis. Such disruptions can interfere with normal embryonic folding and vascular development, resulting in a cascade of anomalies whose severity depends on the timing and extent of the insult.

This developmental perspective highlights the need for further research into the embryological mechanisms and risk factors underlying BSAs in animals. A more holistic understanding of these complex malformations could enhance diagnostic accuracy, inform breeding and management practices, and contribute to comparative models of congenital disorders across species.

## Figures and Tables

**Table 1:** Data for seven dog fetuses, including weight, length, defect size, breed, delivery type, maternal age, litter size, gestation and mating type.

Case	Weight (g)	CRL (cm)	BWD (cm)		Parturition					Breed
				Type	Mother’s Age (years)	Litter Size	Gestation Time	Maternal Parity	Mating	
C 1 ♀	280	13.5	2.9 × 2.9	C-section	3	6	59	P	Natural	Pitbull
C 2 ♂	125	9.8	1.1 ×2.9	Natural delivery	2	7	60	M	Natural	Medium-sized mongrel dog
C 3 ♂	105	8.2	2.9 × 3	C-section	2	3	59	P	Natural	Yorkshire terrier
C 4 ♂	85	7.5	0.6 × 0.6	C-section	3	6	60	M	Natural	Teckel
C 5 ♂	62	11.4	1.5 × 0.9	Dystocia	4	3	60	P	Natural	Yorkshire terrier
C 6 ♂	60	9.5	0.4 × 0.4	C-section	2.5	4	61	UNK	AI	Pincher
C 7 ♂	105	10.1	0.5 × 0.5	Natural delivery	6	5	60	P	Natural	Mongrel dog (teckel-like)

Note: AI, artificial insemination; BWD, body wall defect; CRL, crown–rump length; M, multiparous; P, primiparous; UNK, unknown; ♀, female; ♂, male.

**Table 2:** Summary of the anomalies described in the studied canine cases and diagnosed as body stalk anomalies (BSAs).

Case	BWD	ASA	UCA	LA	AA	CFA	UGA	AB	Diagnosis
C 1 ♀	l-CLO Exposure to the exterior of the spleen, liver along with the gallbladder, pancreas, part of the stomach, and all the intestine except the descending colon and the rectum	S Hypoplasia of S1 and S2, agenesis of S3 and caudal vertebrae. Hypoplasia of both the ilium and the ischium and agenesis of both pubes	DUV. SUA	MLPL	+	−	Persistent urogenital sinus. Genital hypoplasia. Left renal agenesis	−	BSA III SPLBWC
C 2 ♂	l-CLAb JLE	S HV at the T5–T7 level, hypoplasia of left ribs 6, 7, and 8 and agenesis of left ribs 10 to 13. Agenesis of sacral and caudal vertebrae	DUV HLUA	MLPL	−	−	Hypoplasia of the genital tract and the genital organs	−	BSA III SPLBWC
C 3 ♂	r-LAb External exposure of the kidneys, spleen, liver along with the gallbladder, pancreas, part of the stomach, and all the intestine except the descending colon and the rectum	LLRF ES Vertebral block at the point of maximum flexion of the spine and presence of HV at the thoracolumbar junction.	DUV HLUA	ARTL MRTL PRPL ArRPL		SP	Bilateral renal hipoplasia and genital hypoplasia	−	BSA III SPLBWC
C 4 ♂	O JLE	K Presence of HV at level T7–T9 corresponding with the point of maximum flexion of the spine.	DUV	ArPL	+	SP Mouse snout	Hypoplasia of the genital tract and the genital organs	Amniotic adhesion in the right ventrolateral portion of the frontal region. Remnants of the amnion attached to the nostrils	BSA IV SPBWC
C 5 ♂	O JLE	ArCTV Agenesis of the sacrum and the caudal vertebrae.	DUV	ArPL The plantar surface of both extremities is directed medially	+	−	Complete hypospadias	−	BSA IV SPBWC
C 6 ♂	O JLE	MS Presence of a vertebral block T2–T3 and HV at T8 level	DUV	ArLTL	−	ChPa		−	BSA VIII SPBWC
C 7 ♂	O JLE	MS	DUV	ArPL The plantar surface of both extremities is directed dorsally	−	HC		−	BSA VIII SPBWC

Note: AA, anal atresia; AB, amniotic band; Ab, abdominoschisis; ArCTV, arthrogryposis in the cervical and thoracic vertebrae; ArPL, arthrogryposis in the pelvic limb; ArLTL, arthrogryposis in the left thoracic limb; ArRPL, arthrogryposis in the right pelvic limb; ARTL, amelia of the right thoracic limb; BSA, body stalk anomaly; ChPa, cheilognathopalatoschisis; DUV, dispersed umbilical vessels; ES, extreme scoliosis; HC, hydrocephalus; HLUA, hypoplastic left umbilical artery; HV, hemivertebrae; JLE, jejunal loop eventration; K, kyphosis; l-CLA, left caudolateral abdominoschisis; l-CLO, left caudolateral omphalocele; LLRF, left lateral retroflexion; MLPL, meromelia of the left pelvic limb; MLTL, meromelia of the left thoracic limb; MS, mild scoliosis; O, omphalocele; PRPL, phocomelia of the right pelvic limb; r-LAB, right lateral abdominoschisis; S, scoliosis; SP, secondary palatoschisis; SPBWC, spinal body wall complex; SPLBWC, spinal limb body wall complex; SUA, single umbilical artery; UCA, umbilical cord anomaly; UGA, urogenital anomaly; ♀, female; ♂, male.

## Data Availability

The original contributions presented in this study are included in the article. Further inquiries can be directed to the corresponding author.

## Abbreviations

The following abbreviations are used in this manuscript:

AA	Anal atresia
AB	Amniotic band
Ab	Abdominoschisis
ABDC	Amniotic band disruption complex
ArCTV	Arthrogryposis in the cervical and thoracic vertebrae
AI	Artificial insemination
ArPL	Arthrogryposis in the pelvic limb
ArLTL	Arthrogryposis in the left thoracic limb
ArRPL	Arthrogryposis in the right pelvic limb
ARTL	Amelia of the right thoracic limb
ASA	Axial skeletal anomaly
BCH	Bilateral cheiloschisis
BSA	Body stalk anomaly
BWD	Body wall defect
CBWA	Complex body wall anomaly
CFA	Craniofacial anomalies
ChPa	Cheilognathopalatoschisis
CP	Complete palatoschisis
CRL	Crown-rump length
DUV	Dispersed umbilical vessels
ES	Extreme scoliosis
HC	Hydrocephalus
HLUA	Hypoplastic left umbilical artery
HV	Hemivertebrae
JLE	Jejunal loop eventration
K	Kyphosis
LA	Limb anomaly
l-CDLAB	Left caudolateral abdominoschisis
l-CLO	Left caudolateral omphalocele
LLRF	Left lateral retroflexion
MLPL	Meromelia of the left pelvic limb
MLTL	Meromelia of the left thoracic limb
MS	Mild scoliosis
PRPL	Phocomelia of the right pelvic limb
O	Omphalocele
r-Lab	Right lateral abodominoschisis
S	Scoliosis
SPBWC	Spinal–body wall complex
SPLBWC	Spinal–limb–body wall complex
SSBWC	Sternal–spinal–body wall complex
STBWC	Sternal–body wall complex
SUA	Single umbilical artery
UC	Umbilical cord
UCA	Umbilical cord anomaly
UGA	Urogenital anomaly
